# Passive acquisition of anti-*Staphylococcus aureus* antibodies by newborns via transplacental transfer and breastfeeding, regardless of maternal colonization

**DOI:** 10.6061/clinics/2016(12)02

**Published:** 2016-12

**Authors:** Maria Isabel Valdomir Nadaf, Laila Lima, Inês Stranieri, Olga AkikoTakano, Magda Carneiro-Sampaio, Patricia Palmeira

**Affiliations:** I Universidade Federal do Mato Grosso (UFMT), Departamento de Pediatria, Mato Grosso/MT, Brazil; II Faculdade de Medicina da Universidade de São Paulo, Departamento de Pediatria, São Paulo/SP, Brazil; III Hospital das Clínicas, Instituto da Criança, Laboratório de Investigação Médica (LIM-36), São Paulo/SP, Brazil

**Keywords:** *Staphylococcus aureus*, Passive Antibody Transfer, IgG1 and IgG2 Subclasses, Secretory IgA, Antibody Avidity

## Abstract

**OBJECTIVE:**

To investigate the transmission of anti-*Staphylococcus aureus* (*Sa*) IgG, IgG1 and IgG2 via placental transfer and the transfer of IgA via the colostrum according to maternal *Sa* carrier status at delivery.

**METHODS:**

We evaluated anti-*Sa* IgG, IgG1 and IgG2 in maternal and cord sera and IgA in colostrum from a case (n=49, *Sa*^+^) and a control group (n=98, *Sa*^-^).

**RESULTS:**

Of the 250 parturients analyzed for this study, 49 were nasally colonized with *S. aureus* (prevalence of 19.6%). Ninety-eight non-colonized subjects were selected for the control group. The anti-*Sa* IgG, IgG1 and IgG2 levels and the IgG avidity indexes in the maternal and cord sera did not differ between the groups, with a low transfer ratio of anti-*Sa* IgG to the newborns in both groups. The anti-*Sa* IgG2 titers were significantly higher than the IgG1 titers in the maternal and cord sera. Inversely, the transfer ratios were higher for anti-Sa IgG1 compared with IgG2; however, no differences between the groups were detected. The *Sa*-specific IgA levels and avidity indexes in the colostrum were equivalent between groups.

**CONCLUSIONS:**

Maternal *Sa* nasal colonization at delivery is not associated with higher antibody levels in the mother or newborns. The high titers of anti-*Sa* IgG2 found in the cord serum indicate a greater reactivity with non-protein antigens, which may further contribute to the susceptibility to staphylococcal infections at birth. The presence of IgA in the colostrum with avidity to *S. aureus* reinforces the importance of breastfeeding shortly after birth.

## INTRODUCTION

*Staphylococcus aureus* (*S. aureus*) is among the bacteria that colonize the mucous membranes of pregnant women and, soon after birth, their newborns (NB). In the neonatal period, *S. aureus* has been associated with a variety of disorders of varying degrees of severity, such as nosocomial outbreaks of neonatal impetigo 1, omphalitis 2, arthritis and osteomyelitis 3, late neonatal nosocomial or community-acquired sepsis 4,5, and sudden infant death syndrome 6. A systematic review of the literature, with 19 studies and more than 4000 blood culture isolates identified that the most common causes of neonatal bacteremia were S*taphylococcus aureus, Escherichia coli* and *Klebsiella spp.*, which accounted for 55% of culture positive sepsis; and using a meta-analysis to weight for study size, *S. aureus* isolates accounted for 26% of bacterial sepsis cases among neonates 4.

Colonization studies of paired mothers and children have shown that, from birth, children from mothers with an *S. aureus* nasal carriage are more likely to be colonized by this microorganism than children from non-colonized mothers, with a high genomic concordance between the maternal and newborn *S. aureus* strains 7,8.

*S. aureus* that passes across epithelial barriers undergoes phagocytosis and bacterial killing, with a significant involvement of neutrophils 9. Placental transfer of serum IgG and IgA transmission in the colostrum from the mother to her newborn may contribute to the processes of bacterial neutralization and exclusion and the establishment of the intestinal microbiota 10,11. Because newborn neutrophils are characterized by lower chemotaxis, phagocytosis, and oxidative burst responses 5 and the acquired immune response is still being developed, the passive transfer of maternal antibodies may improve the opsonophagocytic capacity of newborns against *S. aureus* 6.

To gain a better understanding of the passive protection provided by the mother, we investigated whether mothers can provide *S. aureus-*specific antibodies to their infants according to maternal nasal carrier status. Because the nature of the antibody transmitted to the newborn, i.e., the predominant antibody subclass or antibody avidity, can interfere with the effectiveness of placental transfer and newborn protection, we evaluated the presence of anti-*S. aureus* antibodies in the maternal and umbilical cord sera or the colostrum and evaluated whether maternal carrier status during delivery influenced the amount and nature of the antibody.

## MATERIALS AND METHODS

### Study population

This was a cross-cohort study of paired parturients and their term newborns with (n=49) and without (n=98) nasal colonization by *S. aureus*. Two hundred and fifty mothers were selected at two maternity wards in Cuiabá, Mato Grosso, Brazil, during the period from August 2011 to September 2012, according to the following inclusion criteria: healthy mothers older than 20 years old with healthy term newborns with an adequate weight for the gestational age and a 5 minute Apgar score >7. The exclusion criteria included placental malformation, chronic disease, severe infectious disease either during pregnancy or during the delivery period and a positive serology for conventional serologic tests.

The samples were collected after informed consent and the approval of the Ethics Committee of the University Hospital (Protocol 936/CEP-HUJM/2010), the Research and Ethics Committee of the Department of Pediatrics of São Paulo University School of Medicine and the Ethics Committee for the Analysis of Projects and Research of the Hospital das Clínicas (Project 316/11). All of the guidelines for human experimentation were followed.

Blood samples and nasal swabs were collected from the mothers shortly before delivery, and the blood samples were collected from the respective newborn umbilical cords immediately after delivery from a large vein on the fetal side of the placenta. The sera were separated, aliquoted and stored at -80°C until use. The colostrum samples were collected approximately 24 hours after delivery. The samples were defatted by centrifugation, and the liquid phase was stored in aliquots at -80°C.

Two control pools were used: a human serum pool prepared with serum from healthy 18-40-year-old blood donors with negative results for conventional serologic tests and a human colostrum pool prepared with colostrum from healthy mothers; both were already available in our laboratory.

### *S. aureus* isolation and identification

Nasal swabs from all of the parturients were placed in Stuart medium (Absorve^®^ CRAL, Cotia, SP, Brazil) for transport and inoculated in mannitol salt agar for 24 hours at 35°C. The identification of *S. aureu*s colony forming units (CFUs) and antimicrobial susceptibility by minimal inhibitory concentration (MIC) were performed by an automated method using Vitek 2 equipment (Biomerieux^®^, Marcy-l'Étoile, France). The *S. aureus* strain used in this study, ISA35, was isolated from community with 99% identity with *S. aureus* and defined as a methicillin-sensitive *S. aureus* (MSSA).

### Total serum IgG and colostrum IgA determination

The total IgG concentrations were measured in the maternal and umbilical cord serum using the immunoturbidimetry technique. The results were expressed in mg/dL. The total IgA antibodies present in the maternal colostrum were measured by ELISA as previously described 12, and the results were expressed in g/L.

### Anti-*S. aureus* IgG and IgA determination

The anti-*S. aureus* (*Sa*) IgG concentrations in the maternal and umbilical cord serum and the anti-*Sa* IgA concentrations in the colostrum were determined by enzyme-linked immunosorbent assays (ELISA) as described by Carbonare et al. 13 with some modifications. In brief, an overnight culture of *S. aureus* grown in BHI broth at 37°C was inactivated, centrifuged and resuspended in a 1% EDAC (N-(3-dimethylaminopropyl)-N′-ethylcarbodiimide hydrochloride, Sigma, St. Louis, MO, USA) solution in distilled water to an optical density (OD) of 0.8 at 540 nm. An aliquot of 100 μl of this suspension was used in each well for coating the microplates (Costar, Cambridge, MA, USA), which were maintained for 16 to 18 hours at 37°C. After blocking with 1% non-fat milk, the samples or pools were incubated in duplicate in four serial dilution steps for 2 hours at 37°C. The plates were incubated with peroxidase-conjugated anti-human IgG or anti-human IgA (Sigma, St. Louis, MO, USA) for 90 minutes at 37°C, and the reaction was developed with 0.4 mg orthophenylenediamine/ml (Sigma, St. Louis, MO, USA) and read at 492 nm. The plates were washed with PBS–0.1% Tween between each step. The anti-*Sa* IgG and IgA concentrations were expressed as arbitrary units (AU/ml) that were obtained by a comparison to the OD values of the serum or colostrum pool, both defined to contain 1000 AU/ml of anti-*Sa* IgG or IgA, respectively.

### Serum anti-*S. aureus* IgG1 and IgG2 subclasses

The microplates were adsorbed with *S. aureus* as described above. After blocking, the paired maternal and umbilical cord serum samples were added in duplicate at four serial dilution steps for 2 hours at 37°C. Biotinylated anti-human IgG1 (555869; BD Pharmingen, San Diego, CA, USA) or IgG2 (555874; BD Pharmingen, San Diego, CA, USA) was used as the secondary antibody, both at dilutions of 1:500, and incubated for 90 minutes. This step was followed by incubation with streptavidin-HRP (554066; BD Pharmingen, San Diego, CA, USA) diluted 1:500 for 90 min. The reaction was completed as previously described. The anti-*Sa* IgG1 and IgG2 titers were determined as the reciprocal of the sample dilution corresponding to an OD of 0.5. We expressed the anti-*Sa* IgG subclass results in titers because it allowed us to compare the results of IgG1 with IgG2 for each sample.

### Anti-*S. aureus* IgG and IgA antibody avidity indexes

The avidities of the anti-*Sa* IgG and IgA antibodies in the maternal and umbilical cord sera and in the maternal colostrum samples were determined by elution with different molarities (M) of potassium thiocyanate (0.0–4.0 M) according to the technique described by Jones et al. 14. The results were expressed as the molarity of potassium thiocyanate necessary to elute 50% of the bound antigen-antibody complexes.

### Statistical analysis

The statistical analyses were performed using GraphPad Prism version 5.0 for Windows (GraphPad Software, San Diego, CA, USA). The significance of the differences between two medians was analyzed using the Wilcoxon signed rank test for related data and the Mann–Whitney test for unrelated data. The significance of the differences between two means was analyzed using the unpaired t-test. The anti-*Sa* IgG, IgG1 and IgG2 antibodies were compared between the maternal and umbilical cord sera using the Spearman correlation coefficient. The significance level was set at *p<0.05* for all analyses. The placental transfer ratios of the total and specific antibodies were defined in each assay as the ratio of the umbilical cord concentrations/maternal concentrations x 100.

## RESULTS

### Characteristics of the study population

Of the 250 parturients selected for this study, 49 were nasally colonized with *S. aureus* and were included in the case group (prevalence of 19.6%). Two hundred and one (80.4%) had negative nasal cultures; of these, we included 98 women, who comprised the control group. The demographic characteristics of the two groups are summarized in Table 1.

### Total IgG concentrations and placental transfer ratios

The descriptive statistical analysis is summarized in Table 2. The maternal sera from the case group had significantly higher IgG concentrations than the maternal sera from the control group. No statistically significant differences were detected in the medians between the umbilical cord serum samples from the case and control groups. The total IgG concentrations in the umbilical cord serum samples from the control group were higher than those in the serum samples from their respective mothers. A significantly lower placental transmission of the total IgG antibodies was detected in the case group than in the control group.

### Anti-*S. aureus* IgG concentrations and placental transfer ratios

The Wilcoxon analyses demonstrated significantly lower anti-*Sa* IgG concentrations in the umbilical cord serum samples from the newborns of the case and control groups than those observed in their respective mothers. The Mann-Whitney analyses revealed no significant differences in the anti-*Sa* IgG concentrations between the mothers and between the umbilical cord serum samples from the case and control groups. The anti-*Sa* IgG transfer ratio was significantly lower in the case group than in the control group (Table 2).

The Spearman correlation analysis revealed high correlation indexes of anti-*Sa* IgG between the paired maternal and newborn sera in the case group (r=0.964, *p<0.00001*) and control group (r=0.885, *p<0.00001*).

### Anti-*S. aureus* IgG1 and IgG2 titers and placental transfer ratios

**Figure 1:**
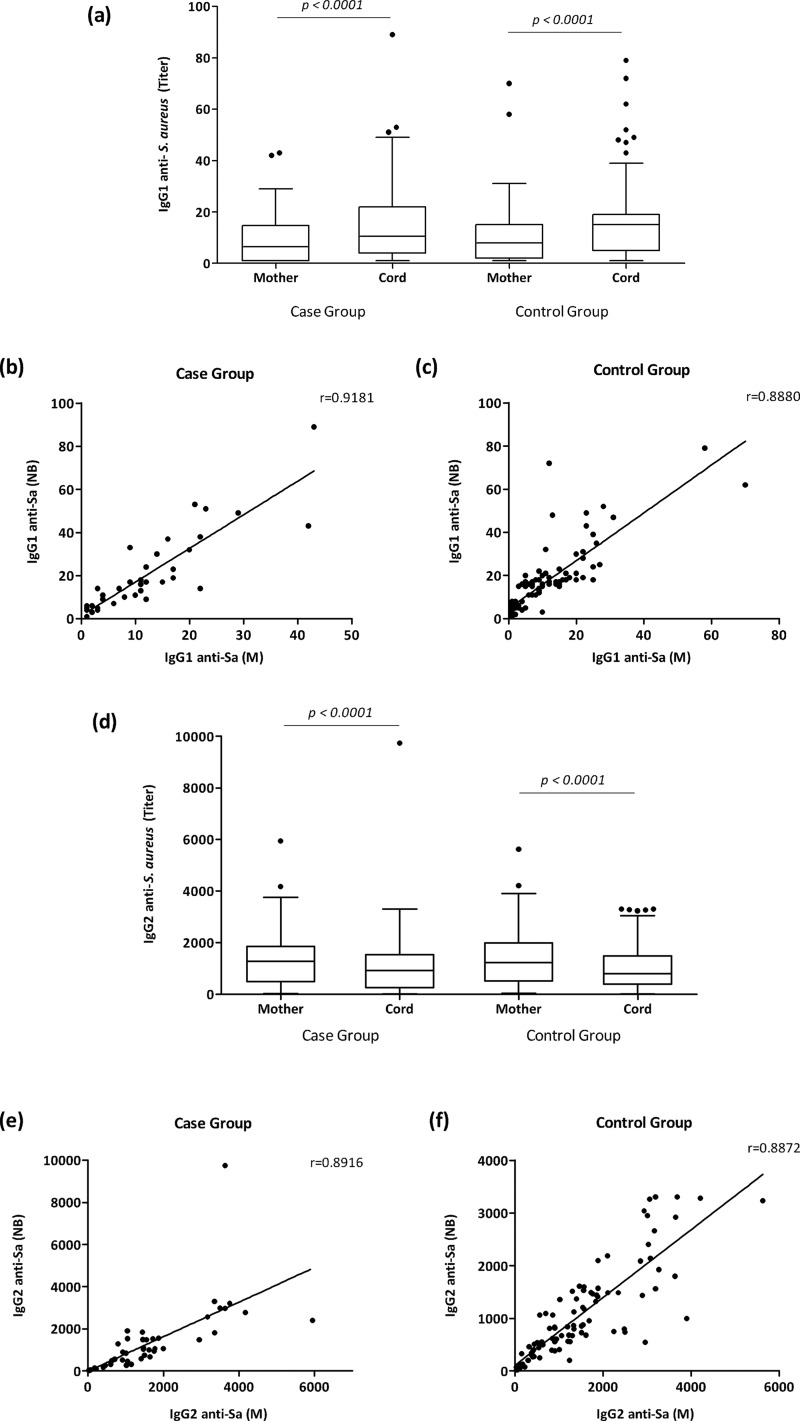
Anti-*S. aureus* IgG1 and IgG2 titers in paired maternal and cord serum samples from the case and control groups. **(a)** Anti-*S. aureus* IgG1 titers and **(d)** anti-*S. aureus* IgG2 titers in the maternal serum and umbilical cord serum samples from the case (n=48) and control group (n=98). In **(b)** and **(c)**, correlation coefficients between paired maternal and umbilical cord serum of anti-*Sa* IgG1 in the case and control group, respectively. In **(e)** and **(f)**, correlation coefficients between the paired maternal and umbilical cord serum of anti-*Sa* IgG2 in the case and control group, respectively. In **(a)** and **(d)**, the box represents the 25^th^–75^th^percentiles, and the median is represented by the line within the box. The whiskers represent the 5^th^–95^th^ percentiles.

The Wilcoxon analyses demonstrated significantly higher anti-*Sa* IgG1 titers in newborns from the case and control groups than those observed in their respective mothers (*p<0.0001*), whereas the analysis of anti-*Sa* IgG2 showed lower levels in the newborns from both groups than in their mothers (*p<0.0001*) (Figure 1a and 1d). When the maternal sera from the case group and the control group were compared, no differences were detected in the anti-*Sa* IgG1 or anti-*Sa* IgG2 titers. The same result was observed between the newborns from both groups (Figure 1a and 1d).

As revealed by the Wilcoxon analyses, the comparison of the anti-*Sa* IgG1 and IgG2 titers showed significantly higher IgG2 titers than anti-*Sa* IgG1 levels in both the maternal and umbilical cord sera from both groups (*p<0.0001*).

The Spearman correlation analysis revealed high correlation indexes of anti-*Sa* IgG1 and anti-*Sa* IgG2 between the paired maternal and newborn serum in both groups (*p<0.00001*) (Figure 1b, c, e and f).

Interestingly, in the case group, the Spearman analysis showed a significant correlation index between the maternal anti-*Sa* IgG levels and the neonatal anti-*Sa* IgG2 titers (r=0.601, *p<0.00001*), and the opposite was also observed, wherein the maternal anti-*Sa* IgG2 titers were correlated with the neonatal anti-*Sa* IgG levels (r=0.544, *p<0.00001*). The same result was observed in the control group when the maternal anti-*Sa* IgG levels and the neonatal anti-*Sa* IgG2 titers were compared (r=0.343, *p<0.001*) and when the maternal anti-*Sa* IgG2 titers and the neonatal anti-*Sa* IgG levels were compared (r=0.364, *p<0.001*). Neither the maternal nor neonatal anti-*Sa* IgG1 titers were correlated with the anti-*Sa* IgG levels or the anti-*Sa* IgG2 titers in either group.

**Figure 2:**
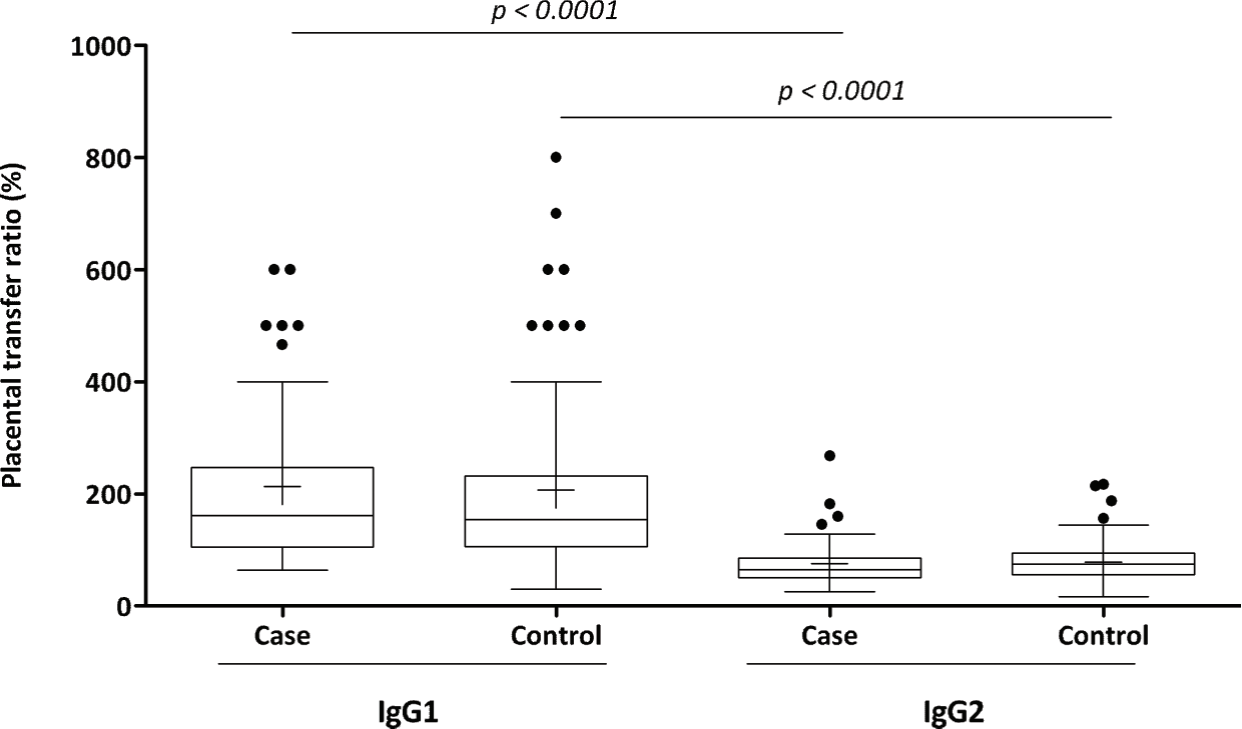
Placental transfer ratios (%) of anti-*S. aureus* IgG1 and IgG2 from case and control groups. Comparison of the data is presented as box and whisker plots. The box represents the 25^th^–75^th^ percentiles, and the median is represented by the line within the box. The whiskers represent the 5^th^–95^th^ percentiles.

No statistically significant differences were detected in the medians of the anti-*Sa* IgG1 placental transfer ratios between the case and control groups or when the placental transfer ratios of anti-*Sa* IgG2 were analyzed. Alternatively, the case and control groups revealed significantly higher anti-*Sa* IgG1 placental transfer ratios than IgG2 (Figure 2).

### Total and anti-*S. aureus* IgA concentrations in the colostrum

Although all of the colostrum samples were collected approximately 24 hours post-partum (range=17.5 - 24.6 hours), the total IgA in the colostra had a large range of variation. Significantly higher total IgA concentrations were observed in the colostrum samples from the case group than in those from the control group (*p<0.05*). The *Sa*-specific IgA concentrations in the maternal colostrum were equivalent between the groups (Table 3).

### Avidity indexes of anti-*S. aureus* IgG and IgA

In both the case and control groups, the avidity indexes of the anti-*Sa* IgG antibodies did not differ between the maternal and umbilical cord serum samples. The same result was observed for the avidity indexes of anti-*Sa* IgA in the colostrum samples, which did not show differences between the case and control groups (Table 4).

## DISCUSSION

The present study revealed similar concentrations of anti-staphylococcal IgG antibodies in the sera of mothers with and without bacterial nasal carriage, suggesting that maternal anti-*Sa* IgG antibodies are not related to colonization with this pathogen. The same effect was observed in the avidity indexes of anti-*Sa* IgG between the maternal or neonatal sera from the case and control groups and between the paired maternal and cord sera. Avidity and antibody specificity are determined by the Fab’ fragment of IgG, while placental transfer is Fc-related; therefore, it was unexpected that antibody avidity could be different between the mother/newborn pairs 15.

*S. aureus* becomes part of the host microbiota within the first hours of life 16, which allows healthy individuals to have high specific antibody levels independent of nasal colonization at the time of measurement 17. Furthermore, anti-staphylococcal IgG and IgA levels and *Sa* antigenic diversity show great individual variability in healthy individuals and in the presence of bacteremia 17,18. This variability has been attributed to strain characteristics, with strain-specific arsenals of evasion mechanisms and differences in the host immune response to this bacterium 19.

In the present study, the placental transfer of total IgG agrees with the current literature that describes positive IgG placental transfer ratios for newborns in healthy full-term pregnancies. However, when the mother has a high content of total IgG or of a specific antibody, the neonatal value usually remains below the maternal value due to the amount of cell surface receptors, and a lower placental transfer ratio is a result of the digestion of unbound IgG molecules by lysosomal enzymes within vesicles 20,21.

The finding that both groups had lower placental transfer ratios of anti-staphylococcal antibodies than expected in an assay performed with whole bacteria prompted us to evaluate specific IgG subclasses. Several studies have demonstrated that the immune response in adults is composed of antibodies reactive to antigenic determinants of bacterial surfaces related to different stages of *Sa* pathogenesis; nevertheless, the highest levels of neutralizing antibodies are predominantly directed to non-protein antigens 17,22.

The higher anti-*Sa* IgG2 than IgG1 titers in maternal and neonatal sera in both groups was unexpected because it is well known that preferential placental transport occurs for IgG1, followed by IgG4, IgG3 and IgG2, for which the FcRn receptors have the lowest affinity 23. In our study, to compare the anti-staphylococcal IgG1 results with those of IgG2 in each sample, the assays were performed under the same conditions, and the subclass results were expressed in titers. As expected, the placental transfer ratio of anti-*Sa* IgG1 was higher than that of IgG2. However, as the maternal anti-staphylococcal IgG1 titers were much lower than those of IgG2, the anti-*Sa* IgG2 titers in neonatal serum were markedly higher than those of IgG1. The predominance of anti-*Sa* IgG2 over IgG1 in the maternal sera could explain the lower placental transfer ratio observed for IgG anti-*Sa* in both the case and control groups. This idea was further reinforced by the significant correlation indexes revealed when the maternal serum anti-*Sa* IgG and neonatal serum anti-*Sa* IgG2 were compared and vice-versa. A study with children with and without otitis media who were vaccinated with *Streptococcus pneumoniae,* another Gram-positive bacterium, showed no differences between groups, and strong correlations were demonstrated between anti-PCP (capsular antigen of *S. pneumoniae*) IgG and the anti-PCP IgG2, as observed in our study 24.

In a study that correlated the protective immune response to a conjugated pneumococcal vaccine, high levels of specific IgG, IgG1 and IgG2 were found, and both IgG subclasses correlated significantly with the opsonophagocytic activity. No significant increases were found in the mean antibody avidities, which were already high in the pre-vaccination samples 25. However, they revealed that neutralization of antibodies directed to the cell wall polysaccharide had no influence on the opsonophagocytic activity of the sera. It has been reported that anti-pneumococcal cell wall polysaccharide antibodies have very little opsonic activity 26.

It is well known that complement poorly lyses the plasma membranes of Gram-positive bacteria because of their thick peptidoglycan layers 27. Thus, it has been argued that antibody-mediated opsonophagocytic bacterial killing is necessary to increase the bactericidal efficiency of MAC against Gram-positive bacteria 28. IgM antibodies and IgG1 and IgG3 subclasses are known to effectively act in these processes, whereas IgG2 shows a much weaker ability. According to our results, this could be an additional limiting factor for newborns to cope with these bacteria 29.

Vaccine trials using the *Sa* capsular polysaccharide 5 and 8 conjugate vaccine revealed that, despite a substantial rise in the post-vaccination anti-CP5 and anti-CP8 antibody concentrations, the *Sa* nasal colonization rates did not significantly change 30.

These results highlight the importance of therapeutic strategies to address Gram-positive bacteria using the induction of immunity by vaccination or passive immunization. These strategies should consider the subclass produced or administered because IgG1 is the main subclass involved in opsonophagocytosis and the interaction of innate and acquired immunity.

Studies aimed at developing vaccines against *Sa* are still performed because carriers have more infections, but these are less severe 31,32, which strongly suggests that some form of immunity has developed during prolonged colonization. The rationale for the development of those vaccines is based on evidence that antibodies directed to toxic shock toxin 33 and α-hemolysin (Hla) reduce the severity of the disease 34, and inhibitors of Hla receptor are also protective 35.

In pregnant women, due to the mucosal immune system and broncoenteromammary axis, secretory IgA produced by the mother against pathogens that colonize mucous membranes is offered to the newborn through the breast milk. This occurs because, once in the nasal site, *Sa* can interact with the nasopharynx-associated lymphoid tissue (NALT), and anti-staphylococcal IgA-producing B lymphocytes can migrate to the mammary gland. The mammary gland then releases specific IgA in breast milk that may help to prevent nasal colonization, since the main characteristic of secretory IgA antibody is to perform immune exclusion by inhibiting colonization and invasion by pathogens 10.

Kawano and Emori 36 assessed total secretory IgA in human milk and found higher IgA concentrations in the colostrum samples at 72 hours post-partum in primiparous women. In the present study, there were more primiparous women in the control group, but we found higher total IgA concentrations in the case group. However, the total IgA concentrations in the colostrum at 24 hours did not differ between primiparous and multiparous women.

Because we found no differences in the anti-*Sa* IgA concentrations and avidity indexes between the groups, nasal colonization was not related to an increase in specific IgA antibodies or avidity indexes in the colostrum.

In conclusion, this study revealed that, in healthy pregnant women, nasal colonization by *S. aureus* at delivery is not associated with higher specific serum IgG concentrations in the mother or in newborns or with higher specific secretory IgA in the colostrum. The high titers of anti-*Sa* IgG2 found in the umbilical cord serum suggest a greater reactivity with non-protein antigens, which may further contribute to the susceptibility to staphylococcal infections at birth. Because newborns are early exposed to *S. aureus* the detection of specific IgA with avidity for this pathogen in the colostrum reinforces the importance of encouraging breastfeeding shortly after birth.

## AUTHOR CONTRIBUTIONS

Nadaf MI, Takano OA and Palmeira P designed the overall study and wrote the manuscript. Nadaf MI and Lima L performed the study, analyzed and interpreted data. Nadaf MI and Takano OA contributed with newborns’ samples and the data collection. Stranieri I performed the *S. aureus* isolation and identification in nasal samples; Carneiro-Sampaio Manalyzed and discussed the results, read and discussed the paper. Palmeira P supervised the performance of experiments, oversaw data analysis and interpretation, co-wrote and edited the manuscript. All authors contributed to the final version of the manuscript and approved it.

## Figures and Tables

**Table 1:** Demographic and obstetric characteristics of the parturients (n=147) according to their nasal *S. aureus* carrier status, Cuiabá-MT.

	*S. aureus* nasal carrier status		
	Case Group (n=49)	Control Group (n=98)	*p* value
Maternal age in years (median)	23	24	*0.3681*
Gestational age in weeks (mean)	39.1	39.1	*0.7942*
Primiparous (n; %)	9; 18.4%	35; 35.7%	*0.0312*
Weight at birth in grams (mean)	3305.0	3275.1	*0.6575*
Placental weight in grams (mean)	635.1	643.8	*0.7035*
Number of cotyledons in each placenta (mean)	10.1	10.2	*0.7093*

**Table 2:** Total IgG, *S. aureus*-specific IgG antibodies and placental IgG transfer ratios in the maternal and umbilical cord serum samples from the case (n=45) and control (n=98) groups.

	Total IgG (mg/dL)		
	Case Group	Control Group	*p* value
**Maternal Serum**	1069.0 [991.0-1116.0]	939.0* [907.0-996.0]	*p<0.01*
**Cord Serum**	1034.0 [1027.0-1138.0]	1041.0* [1044.0-1146.0]	
**Cord/Maternal Ratio (%)**	100.9 [98.9-112.6]	114.1 [112.6-125.9]	*p<0.05*
		**Anti-*S. aureus* IgG (AU/mL)**	
	**Case Group**	**Control Group**	***p* value**
**Maternal Serum**	317.0† [366.6-678.8]	317.4† [409.4-579.9]	
**Cord Serum**	224.7† [243.3-453.8]	264.7† [308.4-429.2]	
**Cord/Maternal Ratio (%)**	69.5 [65.5-76.3]	81.3 [77.3-90.1]	*p<0.05*

The values represent median and confidence intervals (95%).

* Higher total IgG concentrations in the newborns from the control group compared with their respective mothers (*p<0.0001*).

† Lower anti-*Sa* IgG concentrations in the newborns from the case and control groups than observed in their respective mothers (*p<0.0001*).

**Table 3:** Total and *S. aureus*-specific IgA antibodies in the maternal colostrum samples from the case (n=45) and control (n=98) groups.

	Case Group	Control Group	*p* value
**Total IgA (g/L)**	163.3 [152.2 - 216.2]	125.7 [125.8 - 158.3]	*p<0.05*
**Anti-*S. aureus* IgA (AU/mL)**	4019.0 [3747.0 - 7024.0]	5337.0 [6048.0 - 12423.0]	

The values represent median and confidence intervals (95%).

**Table 4:** Avidity indexes of anti-*S. aureus* (*Sa*) IgG in the maternal and umbilical cord serum samples and IgA in the maternal colostrum from the case (n=17) and control (n=18) groups.

	Serum Anti-*Sa* IgG Avidity Index (M)*				Colostrum Anti-*Sa* IgA Avidity Index (M)	
	Case Group		Control Group		Case Group	Control Group
	Maternal	Cord	Maternal	Cord		
Median	1.4	1.3	1.5	1.2	2.5	2.3
Min	0.9	0.9	0.7	0.8	1.3	1.2
Max	3.5	2.9	3.7	3.2	4.0	2.8
CI 95%	1.2-1.9	1.2-1.8	1.3-1.9	1.2-1.9	2.0-2.8	2.0-2.5

* The results were expressed as the molarity (M) of potassium thiocyanate necessary to elute 50% of the bound antigen-antibody complexes.

Min = minimum; Max = maximum; CI 95%= confidence interval 95%.
